# The New Hyperspectral Satellite PRISMA: Imagery for Forest Types Discrimination

**DOI:** 10.3390/s21041182

**Published:** 2021-02-08

**Authors:** Elia Vangi, Giovanni D’Amico, Saverio Francini, Francesca Giannetti, Bruno Lasserre, Marco Marchetti, Gherardo Chirici

**Affiliations:** 1 Dipartimento di Scienze e Tecnologie Agrarie, Alimentari, Ambientali e Forestali, Università degli Studi di Firenze, 50145 Firenze, Italy; elia.vangi@unifi.it (E.V.); giovanni.damico@unifi.it (G.D.); saverio.francini@unifi.it (S.F.); gherardo.chirici@unifi.it (G.C.); 2 Dipartimento di Bioscienze e Territorio, Università degli Studi del Molise, 86100 Campobasso, Italy; lasserre@unimol.it (B.L.); marchettimarco@unimol.it (M.M.); 3 Dipartimento per la Innovazione nei Sistemi Biologici, Agroalimentari e Forestali, Università degli Studi della Tuscia, 01100 Viterbo, Italy; 4 Laboratorio Congiunto ForTech, Università degli Studi di Firenze, 50145 Firenze, Italy; 5 Research Unit COPERNICUS, Università degli Studi di Firenze, 50145 Firenze, Italy

**Keywords:** PRISMA, hyperspectral sensor, hyperspectral imagery, forest types discrimination, separability analysis

## Abstract

Different forest types based on different tree species composition may have similar spectral signatures if observed with traditional multispectral satellite sensors. Hyperspectral imagery, with a more continuous representation of their spectral behavior may instead be used for their classification. The new hyperspectral Precursore IperSpettrale della Missione Applicativa (PRISMA) sensor, developed by the Italian Space Agency, is able to capture images in a continuum of 240 spectral bands ranging between 400 and 2500 nm, with a spectral resolution smaller than 12 nm. The new sensor can be employed for a large number of remote sensing applications, including forest types discrimination. In this study, we compared the capabilities of the new PRISMA sensor against the well-known Sentinel-2 Multi-Spectral Instrument (MSI) in recognition of different forest types through a pairwise separability analysis carried out in two study areas in Italy, using two different nomenclature systems and four separability metrics. The PRISMA hyperspectral sensor, compared to Sentinel-2 MSI, allowed for a better discrimination in all forest types, increasing the performance when the complexity of the nomenclature system also increased. PRISMA achieved an average improvement of 40% for the discrimination between two forest categories (coniferous vs. broadleaves) and of 102% in the discrimination between five forest types based on main tree species groups.

## 1. Introduction

Hyperspectral sensors observe the earth’s surface by simultaneously sampling hundreds of fine narrow contiguous spectral bands with a resolution of up to 0.01 μm in the visible and infrared spectrum. Each pixel in hyperspectral imagery (HSI) corresponds to a spectral vector, which reflects the characteristics of the land cover, making it possible to derive the reflectance behavior of the pixels in the image [1]. The rich spectral information helps to better discriminate surface features and objects than traditional multispectral imaging systems [2]. Hyperspectral sensors are not designed for specific applications, and today we are witnessing the rapid development of hyperspectral image processing technology [3] and spaceborne hyperspectral missions [4]. For this reason, hyperspectral data are increasingly used in several remote sensing fields such as ecology, atmosphere, ocean, agriculture and forestry [5].

The ongoing spaceborne hyperspectral missions were joined in March 2019 by PRISMA (Precursore IperSpettrale della Missione Applicativa), developed and operated by the Italian Space Agency (ASI). The purpose of the mission is to evaluate if the PRISMA sensor can be successfully used for monitoring natural resources and atmospheric characteristics and to evaluate possible new applications for environmental risk management and land observation [6]. The new sensor can be employed for a large number of remote sensing applications. Land cover classification and target detection are some of the most common hyperspectral remote sensing applications [7] and are used to support biodiversity monitoring programs [8]. The underlying assumption in these tasks is that different materials of land cover have unique spectral characteristics [9]. For a “pure” material, these spectral characteristics are called endmembers [10]. Endmembers can be measured in the laboratory, in the field, or can be extracted from remotely sensed imagery. However, when manipulating real scenes, the spectral unicity assumption is difficult to meet because several factors produce noise in an imaging device, e.g., complex atmospheric transmission and interference conditions, as well as the aliasing between adjacent but different materials [9]. An unideal electromagnetic wave transmission environment means that some bands contain less discriminatory information than others [11], and some spectral intervals may not reveal important information for some applications [12]. For these reasons, the large number of hyperspectral bands may affect image classification due to the size, redundancy, and autocorrelation of the data cube.

A detailed description of hyperspectral sensors from various platforms can be already found in several publications [4,12,13,14,15], and here we recall their main characteristics in Table 1.

Several feature selection, spectral feature extraction, and classification methods were developed to cope with the challenging intrinsic nature of hyperspectral data. Some of the traditional approaches for classification include spectral mixture analysis (SMA), multiple endmember spectral mixture analysis (MESMA), and spectral angle mapper (SAM). These methods are based on the assumption that a mixed pixel can be resolved into a group of spectral endmembers, modeled as a linear or a nonlinear combination of these endmembers weighted by their sub-pixel fractional cover [9,16,17]. More recently, machine learning algorithms were used to classify HSI, such as supported vector machine (SVM), random forests (RF), and artificial neural network (ANN) [18]. The latest techniques rely on deep learning, mainly on various convolutional neural network (CNN) architectures [19].

Before starting with classification activities, it is essential to have a better comprehension of the spectral behavior of the different land covers through a discriminatory analysis based on spectral separability criteria. These criteria can be grouped into two categories: probabilistic distance and divergence. Some of the most common probabilistic distances are the Chernoff, Bhattacharyya, and Jeffreys–Matusita (JM) distances, based on the conditional density functions of two given land cover classes. The most common divergence measures are the Kullback–Leibler (KL) and the transformed divergence (TD). These are asymmetrical measures of difference between two probability distributions [11]. All these criteria are pairwise measures based on two-land cover problems. Usually, it is possible to extend the validity of such criteria to the multi-class cases by averaging all pairwise measures [11].

In vegetation studies, and more specifically in forestry applications, several studies have used such criteria to discriminate different tree species or groups of species on a band-by-band basis. Roberts et al. [20] evaluated pairwise forest species separability at leaf to stand scale, by means of hyperspectral data. Vaiphasa et al. [21] were able to identify and distinguish 16 vegetation types in a mangrove wetland in Thailand, through the JM distance. In addition, Dalponte et al. [22,23] adopted JM distance as a separability criterion in the hyperspectral band’s selection task based on a sequential forward floating selection algorithm to classify boreal forest species, with different nomenclature systems. The JM distance was also adopted as a separability measure to reduce the redundancy of the spectral feature extracted from SPOT-5 images, retaining the class separability [24]. Aria et al. [25] evaluated the separability of three land cover classes in the USA, based on the JM and TD criteria, after applying a spectral region splitting method to three AVIRIS hyperspectral scenes. Attarchi and Gloaguen [26] used TD to identify the best combination of features, in an L-band Synthetic Aperture Radar (SAR) and Landsat classification problem, in the mountain environment of the Hyrcanian forest, Iran. TD was also used to assess Sentinel-2’s capability to identify burnt areas in five study areas around the world [27]. More recently, the M-statistic was adopted to assess the capacity of seven spectral bands and 13 spectral indices to distinguish the burned area from four unburned land cover types, in three American states [28].

The degree of separability was also useful in feature selection problems. The bands-selection methods based on separability metrics have shown competitiveness with other methodologies, having the advantages of easy implementation and preservation of the physical interpretation [11,23,29].

This study was aimed at investigating the capabilities of the new PRISMA satellite hyperspectral sensor for the recognition of forest categories through a pairwise separability analysis in two study areas in Italy, on a band-by-band basis. This study was aimed at determining the separability based on two levels of the nomenclature system. First, we tried to separate coniferous vs. broadleaves because such wide forest categories are adopted in the official third level of the European Corine Land Cover (CLC) [30] and are used in the framework of national and international reporting of forest statistics [31,32]. Then, we tried to separate the main groups of tree species (called forest types and representing a fourth level of the CLC nomenclature system) because this nomenclature is used in local forest management and mapping activities [33,34].

In order to understand the improvement of the separability capability of the new PRISMA sensor, we compared the result against the spectral separability of the same classes with the Sentinel-2’s Multi-Spectral Instrument (MSI) that can be considered for the moment a reference benchmark for forest mapping. To the best of our knowledge, this represents the first study aimed at investigating the potential of the new hyperspectral sensor PRISMA in forestry.

### 1.1. Overview of the PRISMA Mission and Instruments

The PRISMA satellite, launched on the 22 March 2019, has a relook time of approximately 29 days. The satellite is in the small size class (830 kg), with an operational lifetime of 5 years. The instruments onboard the platform include a Hyperspectral Imager, able to capture images in a continuum of 239 spectral bands ranging between 400 and 2500 nm, 66 in the Visible Near Infra Red (VNIR) and 173 in the Short Wave Infra Red (SWIR) spectrum, with a spectral resolution smaller than 12 nm, and a spatial resolution of 30 m. Nine bands are acquired in a wavelength overlapping region between the VNIR and the SWIR cube. The spacecraft also carries a 5 m resolution Panchromatic Camera [35]. The images can be acquired in an area of interest spanning from 180° W to 180° E longitude and 70° N to 70° S latitude. The PRISMA hyperspectral sensor is based on prisms as a dispersive element that projects the incoming radiation on a 2-D matrix detector, and the image scanning system is a “Pushbroom” type [36].

In addition, the platform carried a payload data handling and transmission subsystem (PDHT). This unit provides the memory for the temporary storage of the images and ancillary data and oversees the data transmission to the dedicated ground segment station.

The main characteristics of the sensor are listed in Table 2.

PRISMA acquires images on demand, in specific individual locations requested by the users, in a “standard” mode, resulting in a 30 × 30 km scene and a “strip” mode, generating an image 30 km width, and having a maximum length of 1800 km. The combination of hyperspectral and panchromatic products gives the ability to recognize the physical-chemical and geometric characteristics of the target of interest within a scene and can potentially provide major contributions in the field of forest analysis, precision agriculture, water quality assessment, and climate change research [6,37].

**Figure 1:**
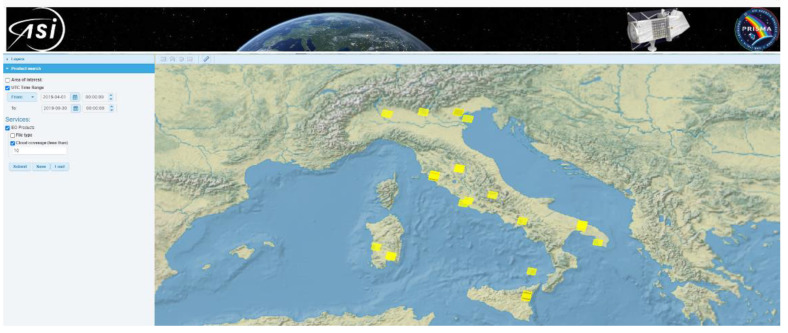
Spatial distribution of all the 23 PRISMA images from the portal prisma.asi.it available in Italy, all of them acquired in 2019 (with cloud cover lower than 10% and acquisition in the vegetative period).

To date, the PRISMA mission has acquired 64,504 images around the globe, of which 58,479 were acquired in 2020 and the remaining in 2019. Searching for images with cloud cover lower than 10% and acquisition during the vegetative period (1 April–30 September) we resulted in only 23 images available in Italy, all acquired in 2019 in 15 different areas (Figure 1).

### 1.2. Preprocessing Levels of PRISMA Hyperspectral Cubes

PRISMA images can be freely downloaded after registration at http://prisma-i.it/index.php/en/ (accessed on 5 February 2021) (Figure 1). They can be released with three levels of preprocessing [35]:

1. Level0: The L0 product contains raw data in binary files, including instrument and satellite ancillary data, like the cloud cover percentage.
2. Level1: The L1 product is a top-of-atmosphere radiance imagery organized as follows: two radiometrically calibrated hyperspectral and panchromatic radiance cubes and two co-registered HYPER and PAN radiance cubes.
3. Level2: The L2 product is divided in:
  - L2B: Atmospheric correction and geolocation of the L1 product (bottom-of-atmosphere radiance);
  - L2C: Atmospheric correction and geolocation of the L1 product (bottom-of-atmosphere reflectance, including aerosol optical thickness and water vapor map);
  - L2D: Geocoding (orthorectification) of L2C.

Levels 1 and 2 are generated on demand and released in the Hierarchical Data Format Release 5 (HDF5). The Level 2 products can be georeferenced with or without ground control points (GCP) according to user preference and GCP availability [6].

## 2. Materials and Methods

### 2.1. Study Areas

**Figure 2:**
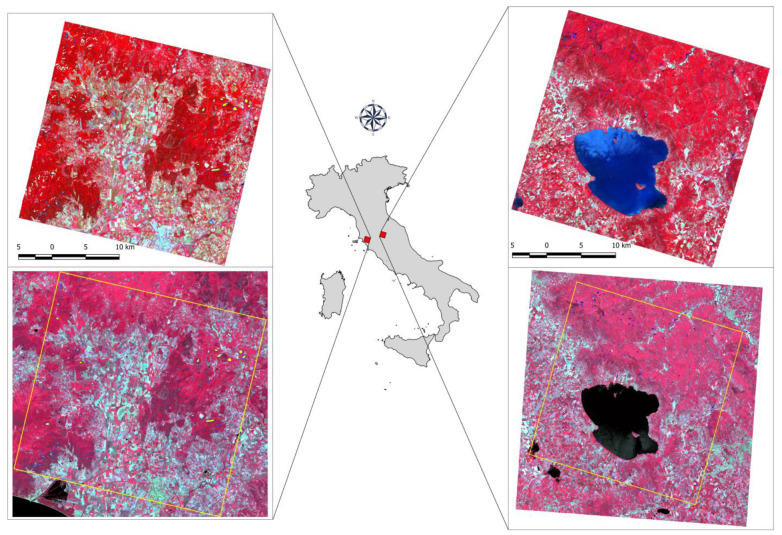
The two study areas. On top: PRISMA false color (RGB 66-35-17); on bottom: Sentinel-2A false color (RGB 8-4-3). In yellow: the boundaries of the PRISMA scene in the Sentinel one.

This study was conducted in two areas located in central Italy (42°53′ N, 11°6′ E and 43°17′ N, 12°13′ E) (Figure 2), each one covering 900 km^2^, just as the PRISMA tiles do. The areas were selected based on the availability of reference data and PRISMA images with a cloud cover < 10% and an acquisition period during the leaf-on vegetation phase.

The first area is in the Province of Grosseto, Tuscany, in the Colline Metallifere, the main and most extensive hilly and mountainous system of the Tuscan Anti-Apennines which includes the city of Grosseto. The area is characterized by gentle slopes (mean slope = 8%) and large altitude differences (from sea level up to 1000 m a.s.l.). The area is dominated by Mediterranean evergreen oaks (*Quercus ilex* L., *Quercus suber* L.) and mesophilic deciduous forests (*Quercus cerris* L., *Quercus pubescens* L., Ostrya carpinifolia Scop., Castanea sativa Mill.). Other tree species include domestic pine (*Pinus pinea* L.), maritime pine (*Pinus pinaster Aito*), and Aleppo Pine (*Pinus halepensis Mill.*). The broadleaves part of the forest was actively managed with coppice clearcut for firewood production.

The second area is in the provinces of Arezzo and Perugia, between the regions of Tuscany and Umbria. The area includes the Trasimeno lake, the fourth largest lake in Italy, and the city of Perugia. The altitude ranges between 170 and 1100 m above sea level, with a steep slope, up to 140%. Broadleaves formations characterized the area, dominated by mesophilic deciduous oaks (*Quercus cerris* L., *Quercus pubescens* L.) and evergreen oaks (mainly *Quercus ilex* L.). Other tree species include Maritime pine (*Pinus pinaster Aito*) and Black pine (*Pinus nigra* A.). The management is less active than in Area 1, but still dominated by coppice for firewood production.

### 2.2. Reference Data

Reference data consist of 161 polygons digitized from the 5 m resolution panchromatic images of the PRISMA satellite to assign the respective third and fourth CLC land cover classes, distributed evenly and proportionally to the abundance of forest types, to ensure that the spectral signatures are as pure as possible.

In the 161 polygons, the forest types were identified on the basis of a local land use and land cover databases of the Tuscan region based on a network of sampling points that are distributed on the basis of an unaligned systematic sampling design [38]. Sampling units are located randomly within 250 × 250 m grid cells for a total of 367.760 points. The nomenclature system we adopted refers to the third level of the Corine Land Cover [26], refined with a fourth level adapted locally (Table 3).

**Figure 3:**
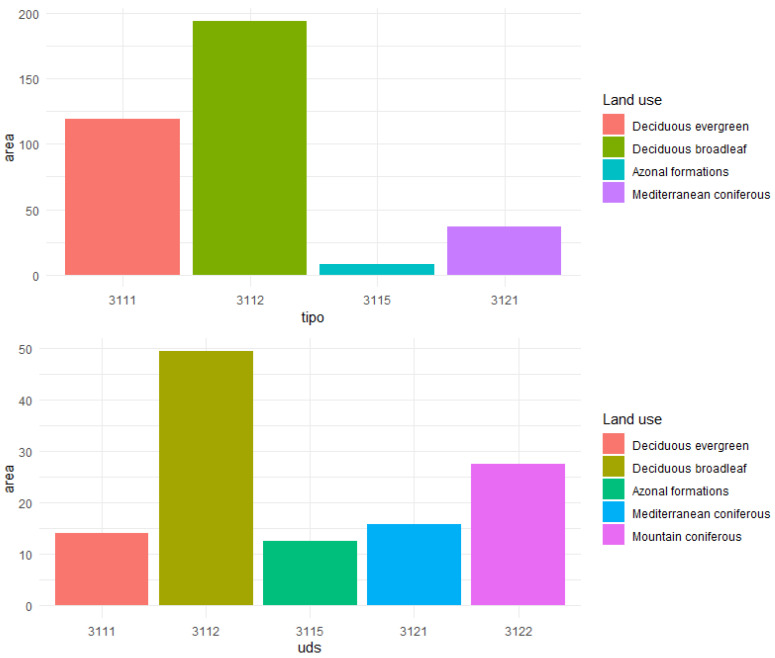
Distribution of forest types according to the reference dataset for the two study areas.

A total of 250 ha (78 polygons) and 220 ha (83 polygons) were acquired in Area 1 and Area 2, respectively (Figure 3).

### 2.3. Remotely Sensed Data

To test the spectral separability of the forest types, we used two PRISMA L2D cloud free scenes acquired on 16 June 2019 and the 4 June 2019, respectively. Each image consists of 239 spectral bands at 30 m spatial resolution ranging between 402 and 2497 nm, with a footprint of 30 × 30 km, atmospherically corrected and orthorectified, provided in he5 format. From the overlapping wavelength region, we retained only the bands from the VNIR cube, for a total of 230 considered spectral bands.

For comparison purposes, we also downloaded for the same areas the Sentinel-2A L2A scenes. The Sentinel-2 images were acquired on the 18 June 2019 and on the 13 June 2019, respectively. All the S2 scenes from the Multi-Spectral Instrument (MSI) have cloud cover <5% and are composed of 13 spectral bands with a spatial resolution of 10, 20, and 60 m depending on the wavelength, ranging between 440 and 2190 nm, with a footprint of 100 × 100 km. S2 images were atmospherically corrected and orthorectified. For this study, the bands b1, b9, and b10 were not used due to their coarse spatial resolution (60 m) and because they are specific to atmospheric characterization and not for land monitoring applications. The remaining bands were resampled to the PRISMA tiles resolution of 30 × 30 m with the nearest-neighbor algorithm.

All the remotely sensed images resulted cloud-free for the forest part of the study areas.

### 2.4. Methods

The PRISMA scenes were first converted in a suitable format by the R package *prismaread* [39], especially developed to import and convert the PRISMA hyperspectral cubes. After the conversion, from the resultant hyperspectral cube, we extracted the reflectance values for every pixel within the 161 reference polygons of the two study areas for each one of the 230 spectral bands. This procedure allows extracting an idealized, pure signature of the spectral classes [40]. These correspond to the full reflectance of pixels exclusively occupied by a single forest type.

The same procedure was repeated for the Sentinel-2A scenes.

A pairwise land cover spectral separability analysis was then carried out for each band of the two sensors. Four commonly used statistical measures were calculated to quantify the two-class separability of the different sensors at each one of the study areas [41,42,43]. The analysis was repeated for both the third and fourth levels of the nomenclature systems. The separability analysis was performed with the R package *spatialEco* [44] for all the possible combinations of each forest type. The statistics were:

1. M-Statistic [45] (M): measures the difference of the distributional peaks of the reflectance values and is calculated as follows:
  (1) $$M=\frac{\mu_{a}-\mu_{b}}{\sigma_{a}+\sigma_{b}}$$
  where *μ_x_* is the mean value for class *x* and *σ_x_* the standard deviation of class *x.* A high M-statistic indicates a good separation between the two classes as the within-class variance is minimized and the between-class variance maximized. The limitation of the M-statistic is that when the means of two classes are equal, the M-statistic will always be zero and cannot accurately reflect the separability.
2. Bhattacharyya distance [46] (B): measures the degree of dissimilarity between any two probability distributions, and is calculated as follows:
  (2) $$B=\frac{1}{8}{\left(\mu_{a}-\mu_{b}\right)}^{T}{\left(\frac{\Sigma_{a}-\Sigma_{b}}{2}\right)}^{-1}\left(\mu_{a}-\mu_{b}\right)+\frac{1}{2}ln\frac{\frac{\Sigma_{a}-\Sigma_{b}}{2}}{\sqrt{\Sigma_{a}-\Sigma_{b}}}$$
  where *μ_x_* is the mean value for class *x* and Σ*_x_* are the covariances. The advantage with respect to the M-statistic is that the Bhattacharyya distance takes into account the class separability due to the covariance difference, expressed in the second term of the equation.
3. The Jeffries–Matusita distance [47] (JM distance): the JM distance is a function of separability that directly relates to the probability of how good a resultant classification will be. It is calculated as a function of the Bhattacharyya distance:
  (3) $$\;JM=\sqrt{2\left(1-e^{-B}\right)}$$
  where *B* is the Bhattacharyya distance.

The JM distance is asymptotic to √2, where values of √2 suggest complete separability. The JM distance can handle data that follow a multivariate normal distribution.

4. Transformed divergence [48,49] (TD): is a maximum likelihood approach that provides a covariance weighted distance between the class means to determine whether spectral signatures were separable:
  (4) $$TD=2\left[1-e^{-\frac{D}{8}}\right]$$
  (5) $$D=\frac{1}{2}tr\left[\left(C_{a}-C_{b}\right)\left(C^{-1}{}_{a}-C^{-1}{}_{b}\right)]+\frac{1}{2}tr[\left(C^{-1}{}_{a}-C^{-1}{}_{b}\right)\left(\mu_{a}-\mu_{b}\right){\left(\mu_{a}-\mu_{b}\right)}^{T}\right]$$
  where *C_x_* is the covariance matrix of class *x*, *μ_x_* is the mean value for class *x*, *tr* is the matrix trace function, and *T* is the matrix transposition function. Transformed divergence ranges between 0 and 2 and gives an exponentially decreasing weight to increasing distances between the classes. As for the JM distance, the transformed divergence values are widely interpreted as being indicative of the probability of performing a correct classification [48].

Lastly, we calculated the percentage variation of the above four metrics obtained by PRISMA with respect to Sentinel-2, for both study areas and the two levels of the nomenclature system. The increment was calculated based on the maximum separability reached in each class pair by the two sensors with the formula:

(6) $$I_{m}=\frac{Max_{P}{}_{m}-Max_{S}{}_{m}}{Max_{S}{}_{m}}\cdot 100$$

where *I_m_* is the percentage increment in separability for the metric *m*, *Max_Pm_* and *Max_Sm_* are, respectively, the maximum value of separability reached by PRISMA and Sentinel-2 for metric *m.*

## 3. Results

**Figure 4:**
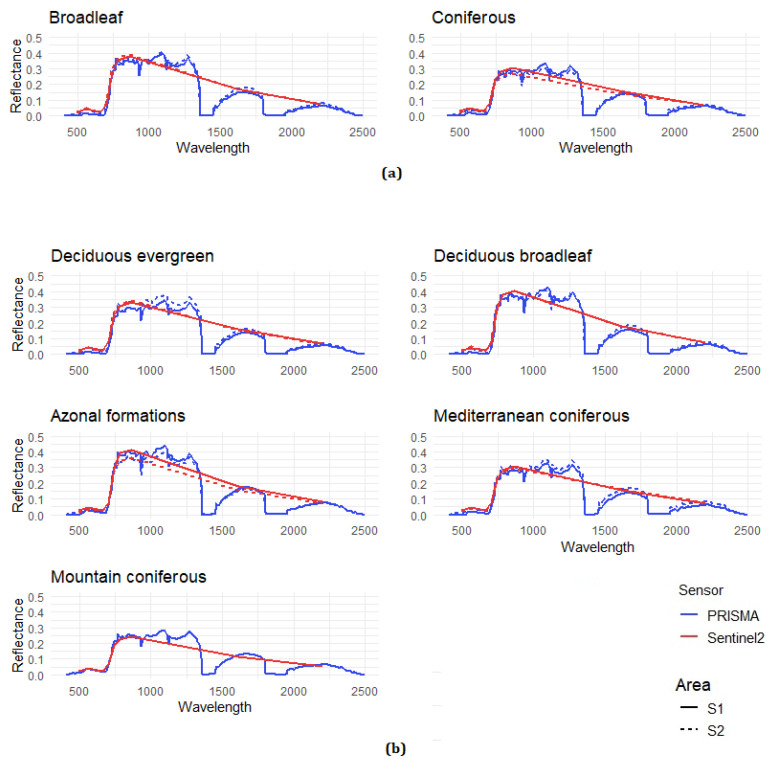
Spectral signatures of the considered forest classes based on PRISMA (blue line) and Sentinel-2 (red line). The solid line for Area 1, the dotted line for Area 2: (**a**) the third level nomenclature system; (**b**) the fourth level.

The spectral signatures derived from PRISMA and Sentinel-2A data are shown in Figure 4. These were calculated as the median reflectance value of every pixel fallen in the specific forest classes of the two levels of the nomenclature system. As expected, the hyperspectral data allowed a more complete and continuous representation of the spectral behavior of the different forest types compared to the multispectral data.

**Figure 5:**
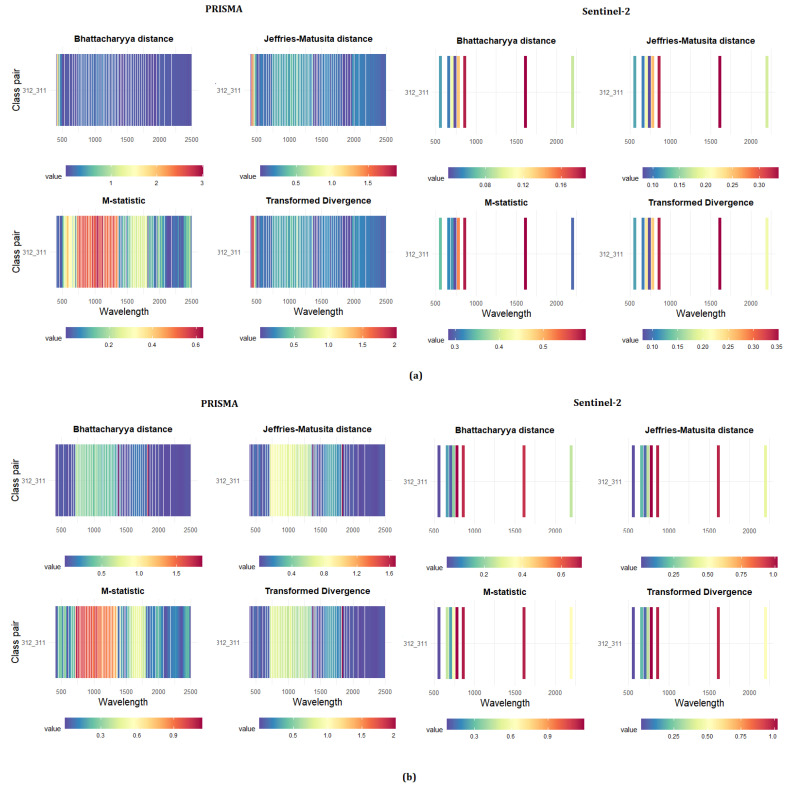
Results of the separability analysis for the third level nomenclature system, divided by statistical metrics and class pairs. The horizontal and vertical axes represent the wavelength and the pairwise vegetation combinations, respectively. The color of each grid cell represents the separability of the corresponding band and class pair, as reported by the legend bar at the bottom of the sub-panels. The higher the value is, the more separable the two classes become: (**a**) Area 1; and (**b**) Area 2.

Based on the spectral signatures extracted within the 161 polygons, the four separability metrics between each pair of classes for each of the 230 bands were calculated for both levels of the nomenclature system, and for both the study areas. The results are shown in Figure 5 and the eight subgraphs represent the four statistical measurements for the two sensors on a band-by-band basis. For the third level, similar trends were observed between the two sensors, but with different results in the two study areas. In Area 1, the PRISMA data allow a better separability for the single class combination, in the visible ranges. The coniferous–broadleaf combination was better distinguished from PRISMA, with a mean separability value of 0.64 occurred in the blue spectrum (between 450 and 503 nm), against a mean value of 0.13 obtained in the SWIR spectrum for Sentinel-2 (1613 nm, band 11). For both sensors, the best metrics for distinguishing the coniferous–broadleaf combination were the transformed divergence and the Jeffries–Matusita distance.

In Area 2, the coniferous and broadleaf were distinguished only in two small portions of the spectrum, near 1380 and 1830 nm, that are not sensed by the Sentinel-2 MSI. In these wavelengths, the PRISMA sensor achieved a mean separability value of 0.75, against 0.43 of Sentinel-2, reached in bands 7, 8, and 11.

**Figure 6:**
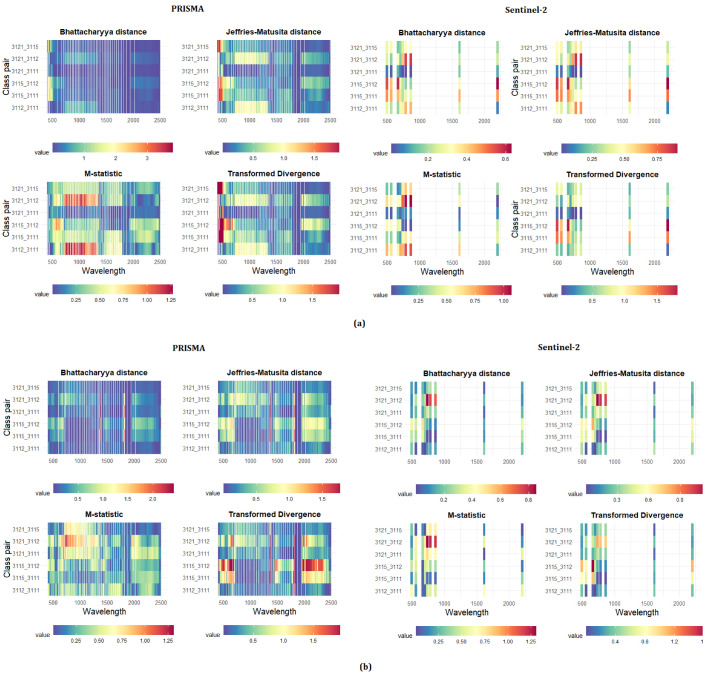
Results of the separability analysis on the fourth level of the nomenclature system, divided by statistical metrics and forest type pairs. Symbology as in Figure 6: (**a**) Area 1; (**b**) Area 2.

The results of the separability analysis on the fourth level of the nomenclature system are shown in Figure 6. In Area 1, the maximum separability value was reached by the PRISMA sensor between 428 and 443 nm, for the pair of Mediterranean coniferous–azonal formations (mean separability value = 0.69), followed by the pairs of azonal formation–evergreen broadleaf (mean separability = 0.68), azonal formation–evergreen broadleaf (mean separability = 0.58), and Mediterranean coniferous—evergreen broadleaf (mean separability = 0.45). The land cover pairs characterized by the least separability were those of Mediterranean coniferous–deciduous evergreen followed by the evergreen broadleaf–deciduous evergreen, with a mean separability value of 0.40 and 0.39, respectively. As for PRISMA, the class pairs better distinguished from Sentinel-2 were Mediterranean coniferous–azonal formation and the evergreen broadleaf–azonal formation, with a mean separability of 0.45 and 0.38, respectively. In Area 2, all the combinations were generally better distinguished by the PRISMA sensor in the NIR and SWIR wavelengths (1373 and 1822 nm), with the Mediterranean coniferous–evergreen broadleaf separability reached the maximum value of 0.74.

In both the study areas, the hyperspectral sensor outperformed Sentinel-2 in the differentiation of all the forest type combinations. Table 4 reports the maximum separability reached by each class pair in all considered metrics. The table also indicates the wavelength at which the maximum separability was reached. The best wavelength range for discrimination proved to be the blue and NIR spectrum in Areas 1 and 2, respectively.

In Table 5 and Table 6, we present the confusion matrix of two-class separability for PRISMA (in blue) and Sentinel-2 (in red), for the third and fourth levels of the nomenclature system, respectively. The cells of the matrix indicate the wavelengths at which the separability for the two classes considered is maximum, according to the average of the four metrics M, JM, B, and TD.

In the third level, the two sensors reached the maximum separability at different wavelengths, in the red–NIR transition zone (called *red edge* for the vegetation spectrum) for Sentinel-2 MSI and in the blue for the SWIR for the PRISMA sensor.

Similar results were obtained for the fourth level. The red edge and NIR regions were best suited for separating the forest types only in Area 2, while in Area 1 the blue channel was particularly adapted to distinguish the azonal formations and deciduous broadleaf. 

**Figure 7:**
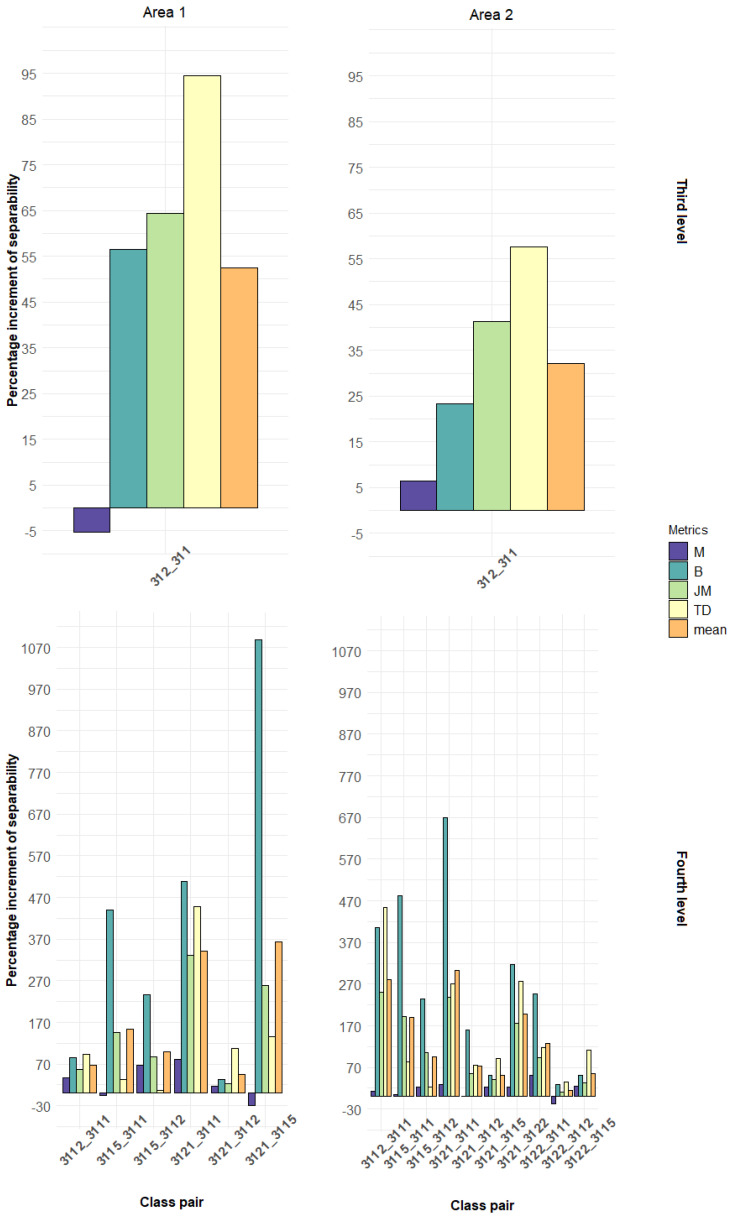
Percentage increment in PRISMA separability in respect to Sentinel-2. On top: the third level; on bottom: the fourth level.

Based on the results of the separability analysis, we calculated the percentage variation of the four metrics obtained by PRISMA concerning Sentinel-2, for both the study areas and the two nomenclature levels (Figure 7).

## 4. Discussion

The separability analysis revealed similar results for the two levels of the nomenclature system and the two study areas. At both levels of the nomenclature system, PRISMA overcame Sentinel-2, but with different scores.

At the third level of the nomenclature system, the broadleaf–coniferous class was well separated by PRISMA, in a narrow range of wavelengths not sensed by the Sentinel-2 MSI, corresponding to the blue (450 nm) and SWIR (1841 nm) bands in Areas 1 and 2, respectively. The differences between the two study areas were most probably due to the characteristics of the terrain. Area 2 presents a more complex topography, with steep slopes and a wide range of elevation, which influence the backscatter of the sensors [50,51]. Therefore, in regions of rapid slope or aspect changes, a large radiometric noise can be expected [51]. In addition, the presence of many shadow areas, due to the forest characteristics, where high and low height trees are mixed, has surely affected the results of the separability analysis and could explain most of the differences between the study areas.

In addition, the effects of varying atmospheric and illumination conditions, due to the time lag between the scene acquisitions, may be of considerable impact. It is worth noticing that the separability was higher in the SWIR channel of PRISMA than in Sentinel-2. Thanks to its spectral resolution and band numbers, hyperspectral images have many advantages in distinguishing the different forest types.

For the fourth level of the nomenclature system, the performance of PRISMA data was even better when compared to Sentinel-2. We found that the separability of forest types was higher in a narrow range of wavelength in the blue channel (430–440 nm) for Area 1, in the NIR-plateau (approximately at 1370 nm), and in the SWIR spectrum at 1822 nm in Area 2. These wavelengths were not sensed by the Sentinel-2 MSI, which primarily relies on the red-edge bands (b6, b7) to discriminate the forest types, because of their high sensitivity to pigment concentration in most leaves and canopies [21]. Our study confirms previous results [17,52,53] where the broadleaves were best separated in the SWIR and NIR spectral ranges, and the coniferous specifically in the SWIR, in bands located directly beyond the water absorption features of the spectrum. This region was also critical for the separation of coniferous–broadleaf combinations, probably due to the differences in leaf water content and total leaf mass between species, which produces a typical species-dependent spectral behavior [53]. The blue channel (450–550 nm), associated with the chlorophyll and other pigments content, was useful for all comparisons between coniferous and broadleaf forest types in Area 1. The better performance of PRISMA at the fourth level of the nomenclature system derived from the combination of the fine spectral resolution and the structural complexity of the forest stand. A forest area with a mixed tree species composition with very similar spectral signatures needs the use of data with finer spectral resolution, while in a forest with few spectrally different species, a coarser spectral resolution can also be used [7]. At the pixel level, the structural complexity of the Mediterranean forest, and the fraction of non-photosynthetic vegetation (that is, bark, branches, wood) affects the extraction of pure spectral signatures.

The low number of field references, observer bias, and time differences between field observation and image acquisition could also influence the results.

Regarding the spatial resolution, it does not appear that the coarser resolution of PRISMA has negatively affected the separability of the classes. As found by Ghosh et al. [54] and Liu et al. [55] a finer spatial resolution is not necessarily better. The former authors obtained better classification accuracy using Hyperion hyperspectral imagery at 30 m spatial resolution than HyMAP imagery at 8 m resolution. Roth et al. [56] demonstrated that 40 and 60 m resolution hyperspectral data can be used to reliably classify most dominant species and plant functional types, in different ecosystem types, including a Mediterranean climate region in California. Moreover, other studies [57,58] have proven that hyperspectral data are less sensitive than multispectral ones in coarsening spatial resolution, due to their greater spectral coverage and finer spectral resolution. In Area 1, the use of the PRISMA sensor improved the recognition between the coniferous and the broadleaves of the third level, in three metrics out of four. In addition, for the fourth level, the fine spectral resolution of the hyperspectral sensor leads to a better separation in all the combinations of forest types. In Area 2, the differences in the performances between the two sensors were generally lower than in Area 1, probably due to the more complex topography and the higher number of forest classes. An in-depth analysis of the slope revealed that here most of the vegetation categories were on a very steep slope, up to 120%, characterized by abrupt changes that caused backscatter interferences and augmented the signal-to-noise ratio of the narrow hyperspectral bands. However, the PRISMA sensor allowed better discrimination in all class pairs, achieving an average improvement among forest types of over 120% in Area 1 and 84% in Area 2, with maximum improvements for some types of 170% in Area 1 and 130% in Area 2.

## 5. Conclusions

In this paper, we evaluated the spectral separability of forest types in two study areas in Italy using the new hyperspectral satellite PRISMA, contrasted against the well-known Sentinel-2 multispectral sensor. The analysis was carried out in the spectral range between 400 and 2500 nm, and with two levels for forest type nomenclature systems. The main findings of this study are:

1. Hyperspectral data were effective in discriminating forest types in both study areas and nomenclature system levels (average normalized separability higher than 0.50 for four out of six classes in Area 1, and nine out of 10 class pairs in Area 2). Only in Area 1 for the third level of nomenclature system the Sentinel-2 MSI was comparable with the PRISMA sensor.
2. The SWIR spectral zone resulted as the most suitable for forest type discrimination. Other remarkable zones were the blue channel (in Area 1) for the broadleaf–coniferous class pair, the red-edge and the NIR-plateau (in Area 2) for most of the considered class pairs. Sentinel-2 relies primarily on the red-edge region (b6, b7) in separating the forest classes.
3. The PRISMA sensor improved the separation between coniferous and broadleaves by 50% in Area 1 and 30% in Area 2. At the fourth level, the average separability of was 120% higher in Area 1 and 84% in Area 2.

This study showed that in the two investigated study areas, the PRISMA hyperspectral sensor had the capability to better discriminate forest types than Sentinel-2 MSI. This was true when the classification requested is for differentiating different forest types, while when aggregated forest classes are used (broadleaves/coniferous). Sentinel-2 MSI can still compete with the hyperspectral sensor. This study also demonstrated that where PRISMA images were not available, Sentinel-2 MSI can be used to separate simple forest classes.

In the future, as the PRISMA data will increase in availability, the new hyperspectral time series can pave the path for more accurate research in plant phenology, forest species classification, the recognition of forest disturbances and change detection studies, making these data very attractive for the forestry sector and beyond. Other fields of expected benefits can be precision agriculture (e.g., crop mapping, crop rotation, crop stress analysis, fertilization), inland and coastal waters (e.g., water quality, chlorophyll monitoring, alga bloom), as well as climate change and environmental research (e.g., desertification, deforestation, vegetation stress, environmental degradation, and hazards).

However, further investigations were needed to explore the full capabilities of the new hyperspectral sensor, for example, testing new algorithms for feature selection and band extraction, the use of vegetation indices, the possibility of automatic segmentation, and object-based classification.

## Figures and Tables

**Table 1:** Main characteristics of spaceborne hyperspectral sensors.

Sensor	Spatial Resolution (m)	Number of Bands	Swath (km)	Spectral Range (nm)	Spectral Resolution	Launch
Hyperion, EO-1 (USA)	30	196	7.5	427–2395	10	2000
CHRIS, PROBA (ESA)	25	19	17.5	200–1050	1.25–11	2001
HyspIRI VSWIR (USA)	60	210	145	380–2500	10	2020
EnMAP HSI (Germany)	30	200	30	420–1030	5–10	Not launched yet
TianGong-1 (China)	10 (VNIR) 20 (SWIR)	128	10	400–2500	10 (VNIR) 23 (SWIR)	2011
HISUI (Japan)	30	185	30	400–2500	10 (VNIR) 12.5 (SWIR)	2019
SHALOM (Italy–Israel)	10	275	30	400–2500	10	2021
HypXIM (France)	8	210	145–600	400–2500	10	2022
PRISMA (Italy	30	240	30	400–2500	10	2019

Legend: VNIR (Visible Near InfraRed); SWIR (Short Wave InfraRed); EO-1 (Earth observation-1); PROBA (Project for On Board Autonom); VSWIR (Visible Short Wave InfraRed); HIS (Hyperspectral Imager); HISUI (Hyperspectral Imager Suite); SHALOM (Spaceborne Hyperspectral Applicative Land and Ocean Mission); HypXIM (HYPperspectral-X Imagery); PRISMA (Precursore IperSpettrale della Missione Applicativa).

**Table 2:** Main characteristics of PRISMA mission 1.

Orbit altitude reference	615 km
Swath/Field of view	30 km/2.77°
Ground Sample Distance	Hyperspectral: 30 m
	PAN: 5 m
Spatial pixels	Hyperspectral: 1000
	PAN: 6000
Pixel size	Hyperspectral: 30 × 30 μm
	PAN: 6.5 × 6.5 μm
Spectral range	VNIR: 400–1010 nm (66 bands)
	SWIR: 920–2500 nm (173 bands)
	PAN: 400–700 nm
Spectral sampling interval (SSI)	≤12 nm
Spectral width	≤12 nm
Spectral calibration accuracy	±0.1 nm
Radiometric quantization	12 bit
VNIR Signal to noise ratio (SNR)	>200:1
SWIR SNR	>100:1
PAN SNR	>240:1
Absolute radiometric accuracy	Better than 5%

**Table 3:** Nomenclature system adopted in this study.

Third Level	Description	Fourth Level	Description
3.1.1	Broadleaf	3.1.1.1	Deciduous evergreen
		3.1.1.2	Deciduous broadleaf
		3.1.1.5	Azonal formation
3.1.2	Coniferous	3.1.2.1	Mediterranean coniferous
		3.1.2.2	Mountain coniferous

**Table 4:** Best separability for each pairwise combination. In blue, the PRISMA sensor; and in red, the Sentinel-2 sensor. The number represents the wavelength where the highest separability was obtained. B for Bhattacharyya distance, JM for Jeffries–Matusita distance. M for M-Statistic, and TD for Transformed Divergence.

Class Pair		PRISMA					Sentinel-2				
		Wavelength	B	JM	M	TD	Wavelength	B	JM	M	TD
Area 1	3112_3111	48	0.81	1.11	1.27	1.11	782	0.44	0.71	0.93	0.73
	3115_3111	236	2.55	140	0.18	2.00	2202	0.47	0.75	0.43	1.47
	3115_3112	464	2.11	1.36	0.22	2.00	2202	0.63	0.93	0.39	1.88
	3121_3111	696	0.90	1.19	0.03	2.00	1613	0.15	0.27	0.05	0.37
	3121_3112	989	0.77	1.07	1.24	1.07	864	0.57	0.87	1.07	0.90
	3121_3115	1156	3.79	1.42	0.18	2.00	782	0.32	0.55	0.69	0.72
Area 2	3112_3111	100	1.00	1.27	0.22	2.00	1613	0.20	0.36	0.63	0.36
	3115_3111	375	2.07	1.35	0.24	2.00	664	0.36	0.60	0.05	1.09
	3115_3112	561	1.71	1.224	0.10	2.00	664	0.51	0.80	0.03	1.62
	3121_3111	791	2.41	1.40	0.20	2.00	740	0.31	0.54	0.79	0.54
	3121_3112	1021	2.21	1.38	0.11	2.00	740	0.85	1.15	1.30	1.15
	3121_3115	1190	0.41	0.67	0.89	0.68	782	0.27	0.47	0.73	0.48
	3121_3122	1480	1.25	1.23	0.15	2.00	1613	0.30	0.52	0.77	0.53
	3122_3111	1985	2.10	1.35	0.24	2.00	740	0.61	0.91	1.10	0.92
	3122_3112	2171	1.75	1.25	0.09	2.00	740	1.35	1.28	1.64	1.48
	3122_3115	2380	0.88	1.17	1.33	1.18	782	0.58	0.88	1.07	0.93

**Table 5:** Third level: best wavelength for two-class separability. In blue, the wavelength extracted from PRISMA; in red, from Sentinel-2.

		Class 2			
		Area 1		Area 2	
		311	312	311	312
Class 1	311	/	864	/	782
	312	450	/	1841	/

**Table 6:** Fourth level: best wavelength for two-class separability. In blue, the wavelength extracted from PRISMA; in red from Sentinel-2.

		Class 2							
		Area 1				Area 2			
		3111	3112	3121	3122	3112	3115	3121	3122
Class 1	3111	/	782	664	/	1613	703	740	740
	3112	814	/	864	/	/	740	740	740
	3115	443	428	782	/	1373	/	782	782
	3121	443	1029	/	/	1373	731	/	1613
	3122	/	/	/	/	1373	1142	1361	/

## Data Availability

Data is contained within the article.

## References

[1] Kumar B., Dikshit O., Gupta A., Singh M.K. (2020). Feature extraction for hyperspectral image classification: A review. Int. J. Remote Sens..

[2] Li S., Wu H., Wan D., Zhu J. (2011). An effective feature selection method for hyperspectral image classification based on genetic algorithm and support vector machine. Knowl. Based Syst..

[3] Acquarelli J., Marchiori E., Buydens L.M.C., Tran T., Van Laarhoven T. (2018). Spectral spatial classification of hyperspec-tral images: Three tricks and a new supervised learning setting. Remote Sens..

[4] Defourny P., D’Andrimont R., Maugnard A., Defourny P. (2018). Survey of Hyperspectral Earth Observation Applications from Space in the Sentinel-2 Context. Remote Sens..

[5] Liu H., Zhang F., Zhang L., Lin Y., Wang S., Xie Y. (2020). UNVI-Based Time Series for Vegetation Discrimination Using Separability Analysis and Random Forest Classification. Remote Sens..

[6] Guarini R., Loizzo R., Longo F., Mari S., Scopa T., Varacalli G. Overview of the prisma space and ground segment and its hyperspectral products. Proceedings of the 2017 IEEE International Geoscience and Remote Sensing Symposium (IGARSS).

[7] Rees G. (2005). The Remote Sensing Data Book.

[8] Travaglini D., Barbati A., Chirici G., Lombardi F., Marchetti M., Corona P. (2007). ForestBIOTA data on deadwood monitoring in Europe. Plant Biosyst..

[9] Hao X., Wu Y., Wang P. (2020). Angle Distance-Based Hierarchical Background Separation Method for Hyperspectral Imagery Target Detection. Remote Sens..

[10] Adams J.B., Smith M.O., Gillespie A.R., Pieters C.M., Englert P.A.J. (1993). Imaging spectroscopy: Interpretation based on spectral mixture analysis. Remote Geochemical Analysis: Elemental and Mineralogical Composition.

[11] Guo B., Damper R., Gunn S.R., Nelson J. (2008). A fast separability-based feature-selection method for high-dimensional remotely sensed image classification. Pattern Recognit..

[12] Staenz K., Held A. Summary of current and future terrestrial civilian hyperspectral spaceborne systems. Proceedings of the 2012 IEEE International Geoscience and Remote Sensing Symposium.

[13] Verrelst J., Romijn E., Kooistra L. (2012). Mapping Vegetation Density in a Heterogeneous River Floodplain Ecosystem Using Pointable CHRIS/PROBA Data. Remote Sens..

[14] Cook B., Corp L., Nelson R., Middleton E., Morton D., McCorkel J., Masek J., Ranson K., Ly V., Montesano P. (2013). NASA Goddard’s LiDAR, Hyperspectral and Thermal (G-LiHT) Airborne Imager. Remote Sens..

[15] Middleton E.M., Ungar S.G., Mandl D.J., Ong L., Frye S.W., Campbell P.E., Landis D.R., Young J.P., Pollack N.H. (2013). The Earth Observing One (EO-1) Satellite Mission: Over a Decade in Space. IEEE Sel. Top. Appl. Earth Obs. Remote Sens..

[16] Yin J., Wang Y., Hu J. (2012). A New Dimensionality Reduction Algorithm for Hyperspectral Image Using Evolutionary Strategy. IEEE Trans. Ind. Inf..

[17] Adams J.B., Smith M.O., Johnson P.E. (1986). Spectral mixture modeling: A new analysis of rock and soil types at the Vi-king Lander 1 site. J. Geophys. Res. Atmos. Solid Earth Planets.

[18] Adam E., Mutanga O., Rugege D. (2010). Multispectral and hyperspectral remote sensing for identification and mapping of wetland vegetation: A review. Wetl. Ecol. Manag..

[19] Kussul N., Lavreniuk M., Skakun S., Shelestov A. (2017). Deep Learning Classification of Land Cover and Crop Types Using Remote Sensing Data. IEEE Geosci. Remote Sens. Lett..

[20] Vali A., Comai S., Matteucci M. (2020). Deep Learning for Land Use and Land Cover Classification based on Hyperspectral and Multispectral Earth Observation Data: A Review. Remote Sens..

[21] Roberts D.A., Ustin S.L., Ogunjemiyo S., Greenberg J.A., Dobrowski S.Z., Chen J., Hinckley T.M. (2004). Spectral and Structural Measures of Northwest Forest Vegetation at Leaf to Landscape Scales. Ecosystems.

[22] Vaiphasa C., Ongsomwang S., Vaiphasa T., Skidmore A.K. (2005). Tropical mangrove species discrimination using hyperspec-tral data: A laboratory study. Estuar. Coast. Shelf Sci..

[23] Dalponte M., Orka H.O., Gobakken T., Gianelle D., Naesset E. (2013). Tree Species Classification in Boreal Forests with Hyperspectral Data. IEEE Trans. Geosci. Remote Sens..

[24] Dalponte M., Bruzzone L., Gianelle D. (2012). Tree species classification in the Southern Alps based on the fusion of very high geometrical resolution multispectral/hyperspectral images and LiDAR data. Remote Sens. Environ..

[25] Zeng W., Lin H., Yan E., Jiang Q., Lu H., Wu S. Optimal selection of remote sensing feature variables for land cov-er classification. Proceedings of the 2018 Fifth International Workshop on Earth Observation and Remote Sensing Applications (EORSA).

[26] Aria S.E.H., Menenti M., Gorte B. Spectral discrimination based on the optimal informative parts of the spectrum. Proceedings of the Image and Signal Processing for Remote Sensing XVIII.

[27] Attarchi S., Gloaguen R. (2014). Classifying Complex Mountainous Forests with L-Band SAR and Landsat Data Integration: A Comparison among Different Machine Learning Methods in the Hyrcanian Forest. Remote Sens..

[28] Huang H., Roy D., Boschetti L., Zhang H.K., Yan L., Kumar S.S., Gomez-Dans J., Li J. (2016). Separability Analysis of Sentinel-2A Multi-Spectral Instrument (MSI) Data for Burned Area Discrimination. Remote Sens..

[29] Ba R., Song W., Li X., Xie Z., Lo S. (2019). Integration of Multiple Spectral Indices and a Neural Network for Burned Area Mapping Based on MODIS Data. Remote Sens..

[30] Chang C.-I., Wang S. (2006). Constrained band selection for hyperspectral imagery. IEEE Trans. Geosci. Remote Sens..

[31] Büttner G., Feranec J., Jaffrain G., Mari L., Maucha G., Soukup T. (2004). The corine land cover 2000 project. EARSeL eProc..

[32] FAO Global Forest Resources Assessment 2010. Terms and Definition. Working Paper 144/E. http://www.fao.org/docrep/014/am665e/am665e00.pdf.

[33] Forest Europe Streamline Global Forest Reporting and Strengthen Collaboration among International Criteria and Indicator Processes. Proceedings of the Joint Workshop.

[34] Vizzarri M., Chiavetta U., Chirici G., Garfì V., Bastrup-Birk A., Marchetti M. (2015). Comparing multisource harmonized forest types mapping: A case study from central Italy. iFor. Biogeosci..

[35] Chiavetta U., Camarretta N., Garfı V., Ottaviano M., Chirici G., Vizzarri M., Marchetti M. (2016). Harmonized forest categories in central Italy. J. Maps.

[36] ASI Prisma Products Specification Document Issue 2.3. http://prisma.asi.it/missionselect/docs/PRISMA%20Product%20Specifications_Is2_3.pdf:.

[37] Loizzo R., Guarini R., Longo F., Scopa T., Formaro R., Facchinetti C., Varacalli G. Prisma: The Italian Hyperspectral Mission. Proceedings of the IGARSS 2018—2018 IEEE International Geoscience and Remote Sensing Symposium.

[38] Fattorini L., Marcheselli M., Pisani C. (2006). A three-phase sampling strategy for largescale multiresource forest invento-ries. J. Agric. Biol. Environ. Stat..

[39] Busetto L. (2020). Prismaread: An R Package for Imporing PRISMA L1/L2 Hyperspectral Data and Convert Them to a More User Friendly Format—v0.1.0. https://github.com/lbusett/prismaread.

[40] Mohan B.K., Porwal A. (2015). Hyperspectral image processing and analysis. Curr. Sci..

[41] Somers B., Asner G.P. (2013). Multi-temporal hyperspectral mixture analysis and feature selection for invasive species mapping in rainforests. Remote Sens. Environ..

[42] Yeom J., Han Y., Kim Y. (2013). Separability analysis and classification of rice fields using KOMPSAT-2 High Resolution Satellite Imagery. Res. J. Chem. Environ..

[43] Hu Q., Sulla-Menashe D., Xu B., Yin H., Tang H., Yang P., Wu W. (2019). A phenology-based spectral and temporal feature se-lection method for crop mapping from satellite time series. Int. J. Appl. Earth Obs. Geoinf..

[44] Evans J., Murphy M.A., Ram K. (2020). Spatialeco: Spatial Analysis and Modelling Utili-Ties. https://CRAN.R-project.org/package=spatialEco.

[45] Kaufman Y., Remer L. (1994). Detection of forests using mid-IR reflectance: An application for aerosol studies. IEEE Trans. Geosci. Remote Sens..

[46] Bhattacharyya A. (1943). On a measure of divergence between two statistical populations defined by their probability dis-tributions. Bull. Calcutta Math. Soc..

[47] Bruzzone L., Roli F., Serpico S.B. (1995). An extension to multiclass cases of the Jefferys-Matusita distance. IEEE Trans. Pattern Anal. Mach. Intell..

[48] Moik J.G. (1980). Digital Processing of Remotely Sensed Images Scientific and Technical Information Branch.

[49] Du H., Chang C.I., Ren H., D’Amico F.M., Jensen J.O. (2004). New Hyperspectral Discrimination Measure for Spectral Characterization. Opt. Eng..

[50] Teillet P., Guindon B., Goodenough D. (1982). On the Slope-Aspect Correction of Multispectral Scanner Data. Can. J. Remote Sens..

[51] Richter R. (1998). Correction of satellite imagery over mountainous terrain. Appl. Opt..

[52] Van Aardt J.A.N., Wynne R.H. (2007). Examining pine spectral separability using hyperspectral data from an airborne sensor: An extension of field-based results. Int. J. Remote Sens..

[53] Fassnacht F.E., Neumann C., Forster M., Buddenbaum H., Ghosh A., Clasen A., Joshi P.K., Koch B. (2014). Comparison of Feature Reduction Algorithms for Classifying Tree Species With Hyperspectral Data on Three Central European Test Sites. IEEE J. Sel. Top. Appl. Earth Obs. Remote Sens..

[54] Ghosh A., Fassnacht F.E., Joshi P.K., Koch B. (2014). Aframework for mapping tree species combining hyperspectral and LiDAR data: Role of selected classifiers and sensor across three spatial scales. Int. J. Appl. Earth Obs. Geoinf..

[55] Liu M., Yu T., Gu X., Sun Z., Yang J., Zhang Z., Mi X., Cao W., Li J. (2020). The Impact of Spatial Resolution on the Classification of Vegetation Types in Highly Fragmented Planting Areas Based on Unmanned Aerial Vehicle Hyperspectral Images. Remote Sens..

[56] Roth K.L., Roberts D.A., Dennison P.E., Peterson S., Alonzo M. (2015). The impact of spatial resolution on the classification of plant species and functional types within imaging spectrometer data. Remote Sens. Environ..

[57] Herold M., Roberts D.A. (2006). Multispectral Satellites—Imaging Spectrometry—LIDAR: Spatial—Spectral Tradeoffs in Urban Mapping. Int. J. Geoinform..

[58] Goodenough D., Dyk A., Niemann K., Pearlman J., Chen H., Han T., Murdoch M., West C. (2003). Processing hyperion and ali for forest classification. IEEE Trans. Geosci. Remote Sens..

